# Supplementary material for: Investigating the influence of physiologically relevant hydrostatic pressure on CHO cell batch culture
Source: Sci Rep. 2021 Jan 8;11:162. doi: 10.1038/s41598-020-80576-8 (PMC7794228; doi:10.1038/s41598-020-80576-8)
Supplement: Supplementary file 1 — Supplementary Information 1. [file 41598_2020_80576_MOESM1_ESM.zip › S1/IgG/Feb17 60 90 standard/standard/20ugml diultion.pdf]

Data File C:\Users\P...s\ChemStation\1\Data\TK_CHO_IgG 2019-02-17 17-28-56\001-2-20ug_ml.D
Sample Name: 20ug/ml

===

Acq. Operator : SYSTEM Seq. Line : 1
Sample Operator : SYSTEM
Acq. Instrument : HPLC-0039 Location : 2
Injection Date : 2/17/2019 5:30:12 PM Inj : 1
Inj Volume : 100.000 µl
Method : C:\Users\Public\Documents\ChemStation\1\Data\TK_CHO_IgG 2019-02-17 17-28-56\CHO_IgG_LC.M (Sequence Method)
Last changed : 2/8/2019 3:17:23 PM by SYSTEM

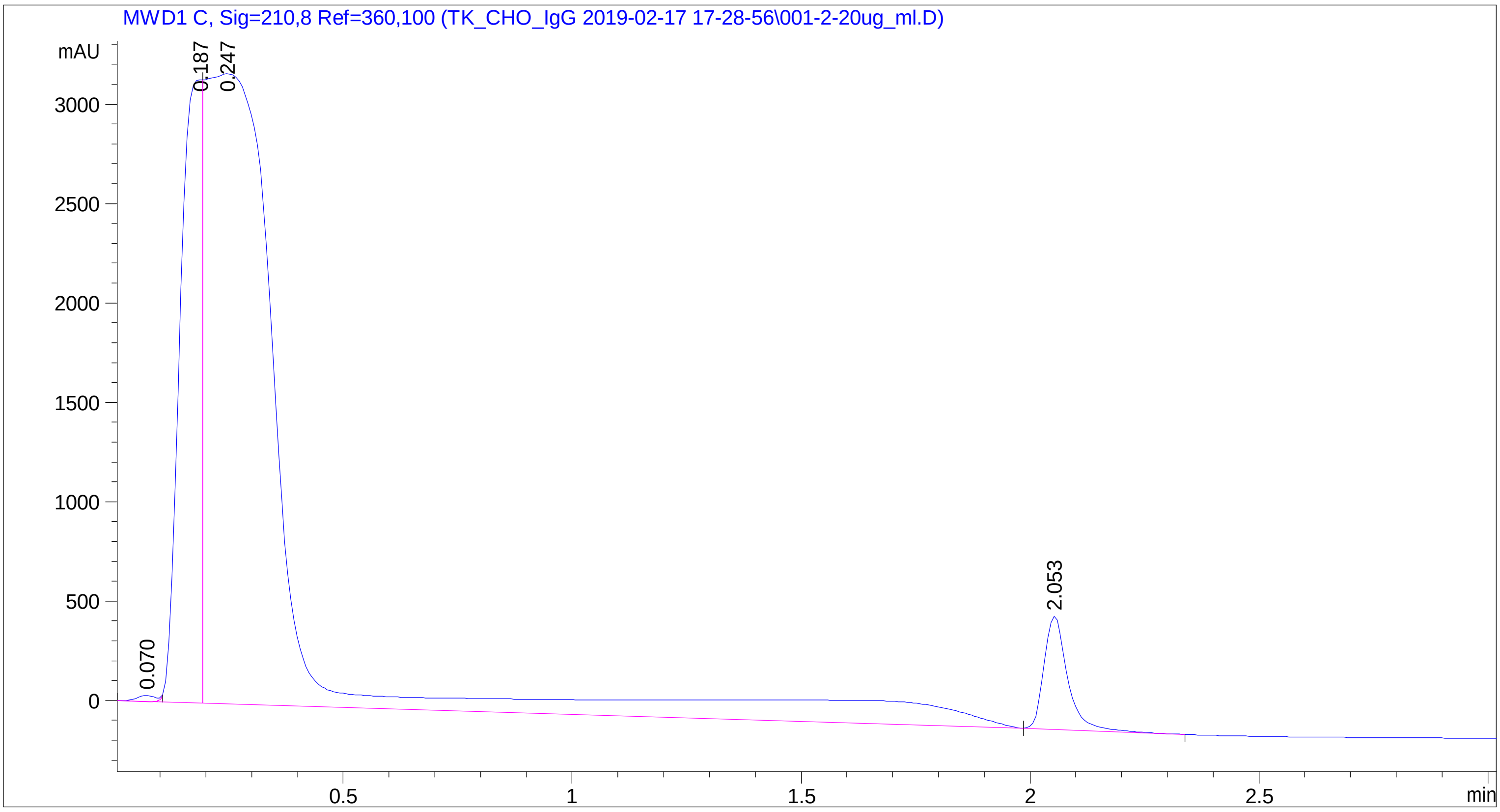

===

Area Percent Report

===

Sorted By : Signal
Multiplier : 1.0000
Dilution : 1.0000
Use Multiplier & Dilution Factor with ISTDs

Signal 1: MWD1 C, Sig=210,8 Ref=360,100

Peak #	RetTime [min]	Type	Width [min]	Area [mAU*s]	Height [mAU]	Area %
1	0.070	BV E	0.0451	82.76296	29.16183	0.1647
2	0.187	VV R	0.0520	1.01748e4	3135.97827	20.2439
3	0.247	VB	0.1757	3.79633e4	3170.10913	75.5322
4	2.053	BBA	0.0558	2040.25720	569.21124	4.0593

Totals : 5.02611e4 6904.46047

===

*** End of Report ***
